# Reactivation of hidden-latent *Brucella* infection after doxycycline and streptomycin treatment in mice

**DOI:** 10.1128/aac.01302-24

**Published:** 2024-12-31

**Authors:** Eugenia Sancho-Sánchez, Kimberly García-Arteaga, Fabio Granados-Chinchilla, Graciela Artavia, Alejandro Alfaro-Alarcón, Andrés Villalobos-Villalobos, Laura Bouza-Mora, Marcela Suárez-Esquivel, Carlos Chacón-Díaz, Caterina Guzmán-Verri, Edgardo Moreno, Elías Barquero-Calvo

**Affiliations:** 1 Programa de Investigación en Enfermedades Tropicales, Escuela de Medicina Veterinaria, Universidad Nacional 504365 , Heredia, Costa Rica; 2 Centro de Investigación en Enfermedades Tropicales, Facultad de Microbiología, Universidad de Costa Rica 495716 , San José, Costa Rica; 3 Escuela de Química, Universidad de Costa Rica, Sede Rodrigo Facio, San Pedro Montes de Oca, San José, Costa Rica; 4 Centro Nacional de Ciencia y Tecnología de Alimentos, Universidad de Costa Rica 27915 , San José, Costa Rica; 5 Departamento de Patología, Escuela de Medicina Veterinaria, Universidad Nacional 504365 , Heredia, Costa Rica; 6 Berlin Institute of Health, Institute of Virology, corporate member of Freie Universität Berlin and Humboldt-Universität zu Berlin, Charité-Universitätsmedizin Berlin 14903 , Berlin, Germany; 7 Departamento de Análisis Clínicos, Escuela de Medicina Veterinaria, Universidad Nacional 504365 , Heredia, Costa Rica; University of California, San Francisco, San Francisco, California, USA

**Keywords:** brucellosis, relapses, antibiotic treatment, streptomycin, doxycycline, cytokines, antibiotic resistance, INF-γ

## Abstract

Brucellosis has therapeutic challenges due to 3%–15% relapses/therapeutic failures (R/TF) after antibiotic treatment. Therefore, determining the antibiotic concentration in tissues, the physiopathological parameters, and the R/TF after treatment is relevant. After exploring different antibiotic quantities, we found that a combined dose of 100 µg/g of doxycycline (for 45 days) and 7.5 µg/g of streptomycin (for 14 days), respectively, achieved therapeutic levels of more than fourfold minimum inhibitory concentrations (MICs) against *Brucella abortus* in the spleen, liver, bone marrow, and plasma of mice, causing minimal pathophysiological effects. After 30 days of infection, mice received antibiotics, and hematological, histopathological, biochemical, and immunological analyses were performed. After antibiotic therapy, the pathological, hematological, immunological, and physiological profiles paralleled those described in human brucellosis. Treatment lowered antibody titers, reduced proinflammatory cytokines, and reduced inflammation in the target organs for a protracted period. No bacteria were detected in tissues 8 weeks after treatment, suggesting complete recovery. However, despite high doxycycline and streptomycin concentrations in tissues, relapses appeared in 100% of the animals after 182 days post-infection, estimated by the bacterial counts and PCR from organs. This proportion contrasts with the 15% R/TF observed in humans after antibiotic treatments. None of the *B. abortus* isolated from relapses showed augmented MICs or mutations coding for antibiotic resistance in chromosomal-relevant regions. We discuss whether our findings constitute a general phenomenon or differences in the exhaustive screening method for bacteria detection related to the murine model. Along these lines, we envision likely mechanisms of bacterial persistence in tissues after antibiotic treatment.

## INTRODUCTION

Brucellosis is a systemic human zoonotic infection caused by intracellular bacteria of the genus *Brucella*, involving many organs and tissues, including the heart and mononuclear phagocytic system, skeletal, reproductive, and central nervous systems (1–3). The complexity of brucellosis lies in its non-pathognomonic protean clinical manifestations, making a clinical diagnosis and management difficult (1–3). The disease’s pathogenic mechanisms and immunobiology are still not fully understood, hindering the development of more effective treatment strategies (4–6). Despite the relative success of antibiotic regimes for a protracted time, which commonly involves the combination of tetracyclines and streptomycin (STR) or tetracyclines and rifampicin (1), induce relapses and therapeutic failures (R/TF) which varied depending on the treatment (7–10). For instance, for the doxycycline (DOX) + STR regime, R/TF rarely exceeds 5%, while for DOX + rifampicin, no more than 15% (11). These R/TF occur most frequently within 6 months after the initial infection, but R/TF may arise years after apparently successful treatment (12, 13).

Studies have shown that antibiotic susceptibility remains constant in *Brucella* isolated before and after antibiotic therapy, indicating that treatment failure is seldom due to acquired resistance (9, 14–16), allowing treatment of the R/TF using the same antibiotic regime as before (7–10, 17). Indeed, the absence of plasmids and lysogenic phages of members of the *Brucella* genus preclude classical antibiotic resistance mechanisms described in other bacteria (18), and just a handful of well-characterized *Brucella* strains with chromosomal antibiotic resistance, mainly rifampicin-resistant genes, have been described (19–21). Moreover, most of these antibiotic-resistant strains are mutants produced in the laboratory for experimental or vaccination purposes, such as *Brucella abortus* RB51 and *Brucella melitensis* Rev1, and many of them are attenuated (20–23).

The mechanisms by which *Brucella* evades antibiotic therapy remain unresolved. It has been proposed that the structure of the *Brucella* cell envelope, the bacterial physiology, and its location within cells of target organs may contribute in part to this phenomenon (5, 24). However, the involvement of various cells, tissues, and organs in brucellosis and the antibiotic concentrations in these sites have barely been investigated, and most research has been based on empirical and comparative observations in clinical surveys (1, 11).

The murine model for antibiotic therapy against brucellosis has been used to estimate the evolution of *Brucella* organisms in the spleen, the course of the antibody response, and the cytokine responses after treatment (25–27). However, no studies have been recorded for determining the optimal concentration of a combined treatment of tetracyclines (e.g., DOX) and STR in the organs to treat brucellosis, estimate R/TF, evaluate hematological, histological, immunological, and organ function parameters after antibiotic treatment for a protracted period. Previously, we determined the concentration of DOX and STR in the mice plasma, liver, spleen, and bone marrow of mice following the regime recommended for brucellosis treatment (28). Here, we have determined in experimentally *B. abortus* infected mice the antibiotic concentration in the targeted organs and evaluated several immunological, hematological, histopathological, and functional parameters after treatment with the most widely used and preferred antibiotics for human brucellosis treatment. Despite high DOX and STR concentrations in tissues, relapses appeared in 100% of the animals, arguing for hidden-latent *Brucella* infections.

## MATERIALS AND METHODS

### Minimal inhibitory concentration determination

*B. abortus* 2308W expressing red fluorescent protein from *Discosoma coral* (*B. abortus*-RFP), provided by Jean-Jacques Letesson (University Notre-Dame de la Paix, Namur, Belgium), was used in all experiments. Minimum inhibitory concentration (MIC) assays were performed on the *B. abortus* strain using DOX (Sigma-Aldrich) and STR (Sigma-Aldrich) (ranging from 0.5 to 250 µg mL^−1^) as described (29).

### Antibiotic quantification in tissues and plasma

The concentration of each antibiotic in tissues and plasma was estimated after intraperitoneally treating mice with DOX (at 50, 100, or 200 µg g^−1^) or STR (at 7.5, 15, or 30 µg g^−1^) every 12 h for 1 week. After 12 h of the last treatment, the antibiotics were quantified in the liver, spleen, bone marrow, and plasma as described (28) with some modifications. Briefly, removed organs were freeze-dried for 24 h. Then, they were macerated and weighed. Spleen, liver, and bone marrow samples were processed for antibiotic extraction with 200 µL of borate buffer, 150 µL of acetonitrile, and 150 µL of methanol. Plasma samples (50 µL) were treated with 200 µL of borate buffer, 125 µL of acetonitrile, and 125 µL of methanol. Extracted samples were analyzed using chromatographic analysis, as described below.

### Reagents for antibiotic quantification

9-Fluorenylmethoxycarbonyl chloride (FMOC), acetonitrile (ACN), and methanol (MeOH), borate buffer (50 mmol L^−1^, pH = 10), trifluoroacetic acid (TFA), dimethyl sulfoxide (DMSO), magnesium acetate tetrahydrate (Mg(CH_3_COO)_2_·4H_2_O), were all chromatographic/ACS grade (or above) and acquired from Sigma-Aldrich (EMD Millipore, Burlington, MA, USA). Ultrapure water (type I, 0.055 μs cm^−1^ at 25°C, 5 µg L^−1^ TOC) was obtained using an A10 Milli-Q Advantage system and an Elix 35 system (EMD Millipore, Burlington, MA, USA).

### Chromatographic analysis

Chromatographic separations were performed using an Agilent Technologies LC system equipped with a high-resolution column (150 × 4.6 mm, 3.5 µm), a 1260 infinity quaternary pump (61311C), column compartment (G1316A), an automatic liquid sampler module (ALS, G7129A), and a fluorescence detector (G1321A) (Agilent Technologies, Santa Clara, CA, USA). Data were analyzed using OpenLab ChemStation (version LTS 01.11, Agilent). A 20 µg mL^−1^ antibiotic standard was used to set a response factor to quantify the amount of antibiotic in the tissues and plasma. Hence, Response Factor = Peak Area_standard_/Concentration_standard_; Sample/Unknown Concentration = Peak Area_sample_/Response Factor.

### Determination of STR using pre-column derivatization

Two hundred microliters sample extract, 190 µL of a borate buffer, 10 µL FMOC solution (i.e., 10 mg FMOC was dissolved in 500 µL ACN and made up in borate buffer in a 10 mL volumetric flask), 100 µL ultrapure H_2_O were sequentially mixed in high-performance liquid chromatography (HPLC) ready-to-inject vial (2 mL, Type 1 borosilicate amber glass, PTFE/silicone screw cap and septa, Santa Clara, CA, USA). STR was determined by adapting a previously reported method (30) to detect a similar antimicrobial agent (31). Briefly, the mobile phase was a mixture of ACN (phase A) and H_2_O (phase B), and it was delivered to the HPLC column at a flow rate of 1 mL min^−1^. The run time was 25 min per analysis. A gradient elution was employed: 0–11 min, to 78% A; 11–11.1 min, to 100% A; hold for 10 min; 20–20.1 min, to 78% A; hold for 5 min. Detection was performed at excitation and emission wavelengths of 260 and 315 nm, respectively.

### Determination of DOX using post-column derivatization

Similarly, a modified version of the proposed method (32) was used to assess DOX residues in murine tissue and plasma. Briefly, a gradient of ACN (phase A) and 20 mmol L^−1^ TFA in H_2_O (phase B) was used as a mobile phase at a flow rate of 0.7 mL min^−1^: 0–10 min: 15% A, 85% B; 10–15 min: 60% A, 40% B. Fluorescent derivatives of doxycycline were generated with DMSO and 0.1 mol L^−1^ magnesium acetate at a flow rate of 0.15 mL min^−1^ at 30°C using a 1.4 mL coil, using a derivatization system (Delta Series Pinnacle PCX, Pickering Laboratories, Mountain View, CA, USA). These derivatives emit light at 473 nm after excitation at 399 nm.

### Infection of mice, antibiotic treatment, and bacterial load quantification

CD1 mice were supplied by the Servicio Nacional de Salud Animal Costa Rica (SENASA). Female mice (18–22 g) were housed under specific pathogen-free conditions and kept in cages with food and water *ad libitum* under biosafety containment conditions. Mice were infected by the intraperitoneal route (i.p.) with 10^4^ colony-forming units (CFU) of *B. abortus-*RFP, and spleen weights and bacteria numbers in different organs were counted as described elsewhere (33). After day 31 post-infection, groups of mice were either treated with phosphate-buffered saline (PBS) (mocked-treated mice) or with STR (7.5 µg g^−1^) for 15 days and DOX (100 µg g^−1^) for 45 days until day 75 post-infection. Mocked mice received 0.1 mL of PBS for 45 days.

### *Brucella* detection in tissues by qPCR

To enrich *Brucella* organisms in tissues, macerated organs in sterile conditions were inoculated in Oxoid Signal Blood Culture Broth and incubated for 24 h at 37°C. DNA was extracted from the cultures using the Wizard Genomic DNA Purification Kit (Promega) as recommended by the fabricant and subjected to quantitative PCR (qPCR) screening as described (34), with some modifications. Briefly, the Master Mix was prepared by combining 12.5 µL of Platinum qPCR Super Mix, 0.5 µL of forward primer (IS421), 0.5 µL of reverse primer (IS511), 0.25 µL of Taqman TAMRA Probe (10 µM), and 9.25 µL of nuclease-free water per reaction, along with 2 µL of DNA sample. DNA was amplified using the Rotor-Gene Q (QIAGEN) thermocycler with the following settings: 95°C for 10 min, 95°C for 15 s for 35 cycles, and 60°C for 1 min for 35 cycles. Each qPCR reaction included a positive DNA control extracted from reference strain *B. abortus* 2308W. In addition, we included a no template control (nuclease-free water) and negative biological control (DNA extracted from a sample known to be free of *Brucella*) as negative controls.

### *Brucella* detection in bone marrow by immunofluorescence

Bone marrow cells were isolated from the tibiae and femora of infected mice by flushing the bones with Hank’s Balanced Salt Solution (without calcium and magnesium) (Sigma). The resulting cell suspension was centrifuged using a Cytospin 2 centrifuge (Shandon) to deposit cells onto slides. The slides were then mounted using ProLong Gold Antifade reagent with DAPI (Thermo Fisher Scientific). Fluorescence microscopy was performed using a Nikon ECLIPSE 80i microscope. For each slide, at least 50 fields of view were examined to ensure thorough analysis. Bone marrow cells isolated from noninfected mice served as negative controls, while cells collected 30 days post-infection were used as positive controls. The presence of intracellular *B. abortus*-RFP within the bone marrow cells was considered a positive result. Photomicrographs were captured using the appropriate fluorescence filter channels, and images were processed and merged using Photopea Online Photo Editor to visualize the co-localization of DAPI and RFP signals.

### Histopathology and hematological profile

For histopathological studies, the spleen and liver were fixed in 10% neutral buffered formalin, processed, and stained with hematoxylin and eosin stain (35). Immunoperoxidase staining of *Brucella* organisms in spleen sections was performed as described previously (36). The histopathology was performed by a certified pathologist who performed all histopathological assays in a single-blinded setting. The histopathology score was estimated by quantifying the granulomatous inflammation induced by *Brucell*a using a semiquantitative analysis and scored as negative (zero) to severe (four) (37–39), as previously described (40). A complete hematological analysis and platelet counts in the blood of mice (collected in EDTA) were performed in an automated VetScan HM5 Hematology Analyzer, following the manufacturer’s instructions.

### Cytokine and antibody quantification

For the quantitative determination of INF-γ, TNF-α, MCP-1, IL-6, IL-12, and IL-10, the serum from mice was collected, and the concentration of cytokines was measured using the Cytometric Bead Array Mouse Inflammation Kit according to the manufacturer’s specifications (BD Biosciences). For antibody titer determination, the sera of mice were tested by microagglutination in a 96-round-bottom well plate as described before (41).

### Evaluation of liver and kidney function

Kidney and liver function tests in plasma were conducted using a Spinreact device (model Spin200e) at the Clinical Analysis Laboratory of the School of Veterinary Medicine at the National University. All determinations were performed according to the manufacturer’s specifications using dedicated lines in the analyzer. For blood urea nitrogen, the kinetic urease-GLDH method was used. For creatinine, the colorimetric Trinder method was employed. The enzymes ALT (GPT) and AST (GOT) were determined using the UV enzymatic IFCC method. The colorimetric Biuret method was used for total proteins, and the colorimetric Bromocresol Green method was employed for albumin (42).

### Whole-genome sequencing and analysis

Whole-genome sequencing (WGS) was performed using the reference strain for infection (*B. abortus-*RFP), one bone marrow isolated from the infected/untreated group, and one bone marrow isolated from the infected/treated relapse group. Genomic DNA was extracted as described above, and its integrity was checked using gel electrophoresis. The DNA was sent to the sequencing facility MicrobesNG (Birmingham, UK) for WGS. Standard sequencing service was performed on the Illumina sequencing platform for all samples. Raw reads were aligned and mapped by bwa v. 0.7.17 (43) and SMALT v.0.5.8 to the reference genomes *B. abortus* 9-941 (GCA_000008145.1) and *B. abortus* 2308W (GCA_000182625.1) with an average mapping of 99.07%. Single nucleotide polymorphisms (SNPs) were retrieved by snp-sites v. 5.0.1 (44) and Samtools/mpileup (45) to determine differences between the isolates.

### Workflow timeline and statistics

**Fig 1:**
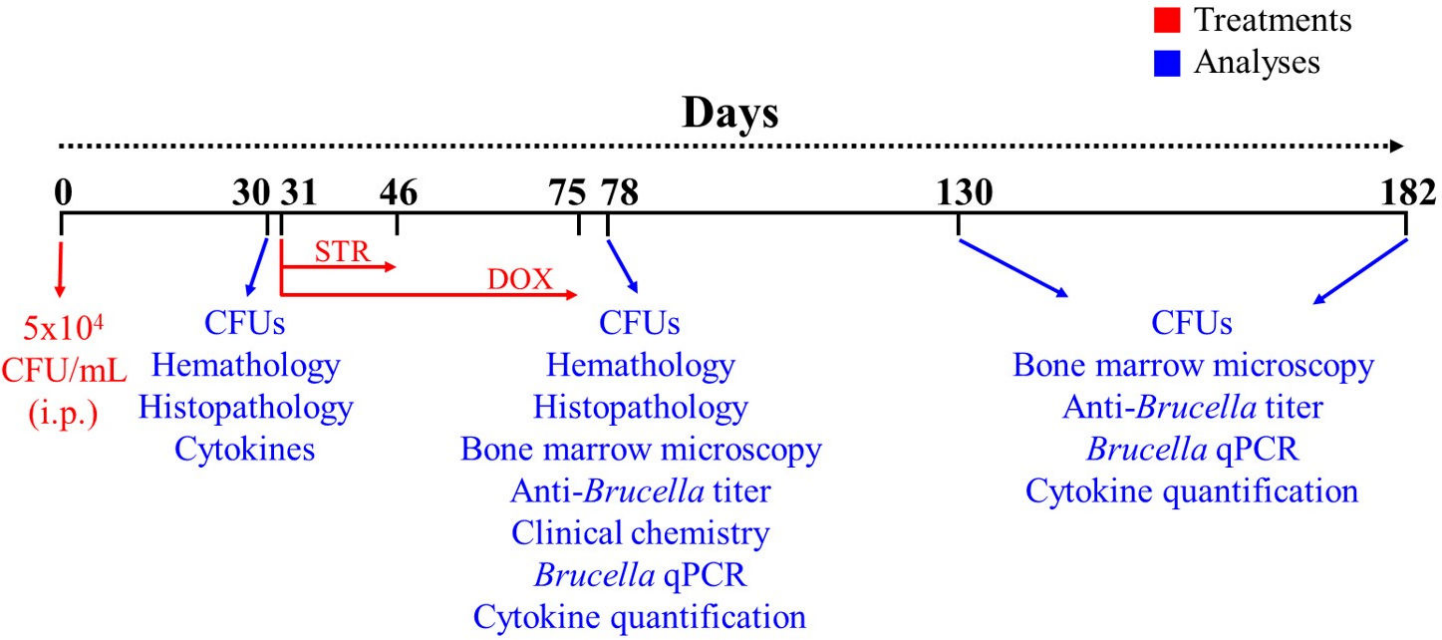
Workflow timeline. Mice were infected with 10^4^ CFU of *B. abortus* 2308. After 30 days post-infection, analyses (blue color) were performed before the antibiotic. At day 31 post-infection, mice were treated with STR (7.5 µg g^−1^) for 15 days and DOX (100 µg g^−1^) for 45 days until day 75 post-infection (red color). At 78, 130, and 183 days post-infection, additional analyses (blue color) were performed.

A schematic representation of the workflow timeline (Fig. 1) was used to evaluate the different parameters of mice after *B. abortus* infection and antibiotic treatment. A one-way analysis of variance, followed by Dunnett’s test, was used to determine the statistical significance of the different assays. The GraphPad Prism software (version 8.0.1) was used for statistical analysis.

## RESULTS

### MIC and antibiotic quantification in tissues and plasma

**Fig 2:**
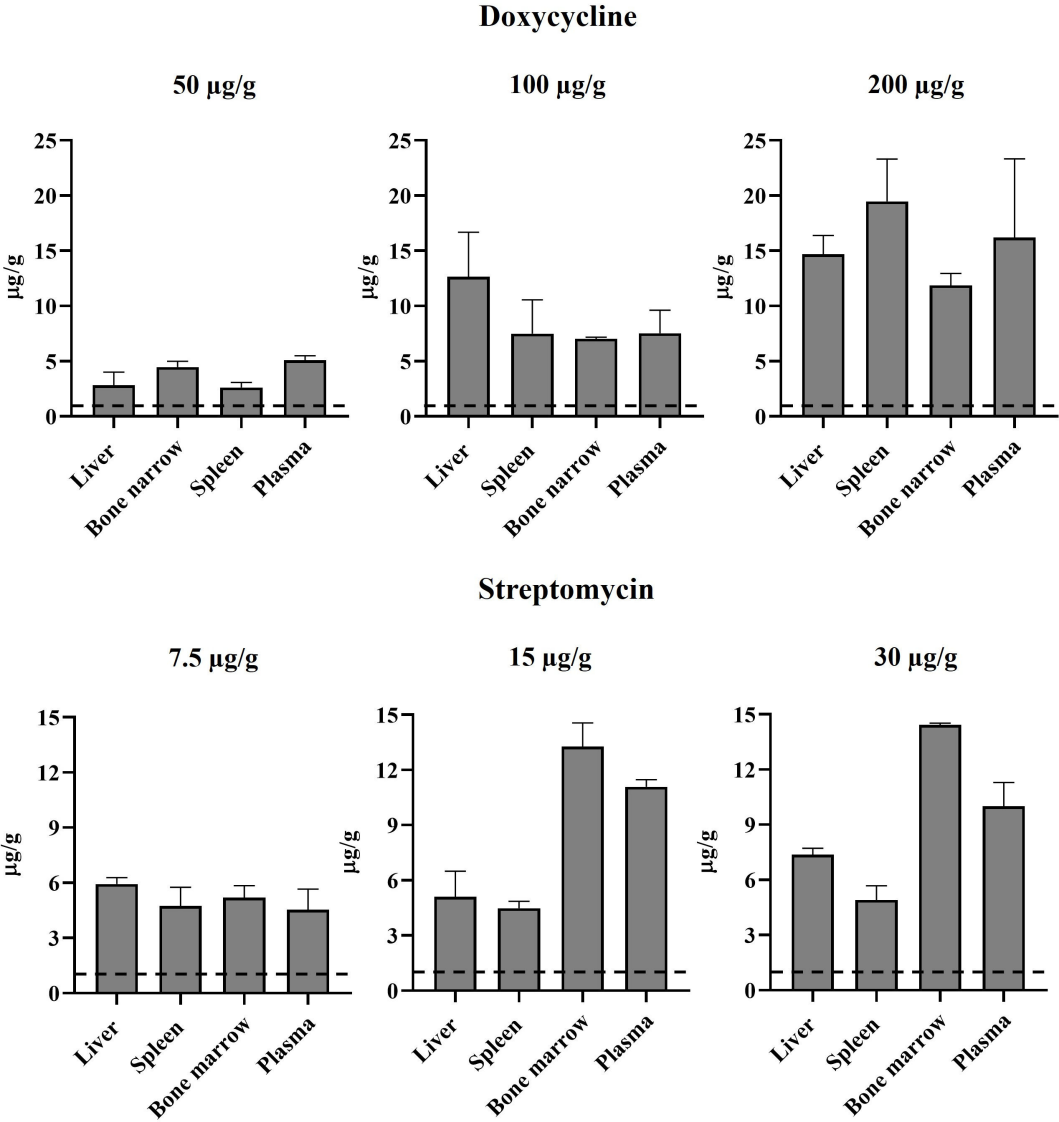
Antibiotic quantification in tissues. Mice were treated intraperitoneally every 12 h with 50, 100, or 200 µg g^−1^ of DOX or 7.5, 15, or 30 µg g^−1^ of STR for 1 week. After 12 h of the last treatment, the antibiotics were extracted and quantified in the liver, spleen, bone marrow, and plasma as described. Extracted samples were analyzed by chromatographic analysis. Dashed lines correspond to each antibiotic’s MIC calculated *in vitro*.

To select an appropriate antibiotic protocol to treat mice infected with *B. abortus*, we first determined the MIC of DOX and STR against reference strain *B. abortus*-RFP. MIC assays were repeated thrice and defined as 0.5 and 1 µg mL^−1^ for DOX and STR, respectively. We then identified the least concentrated antibiotic dosage that reached a therapeutic concentration (at least four times higher than the MIC) (46) in the spleen, liver, bone marrow, and plasma. Therefore, we treated mice with various doses of DOX or STR every 12 h for 1 week. Then, the antibiotic concentration was quantified in tissues and plasma 12 h after the last administration. The lowest doses reaching significant therapeutic concentrations (more than fourfold) in all evaluated tissues and plasma corresponded to 100 and 7.5 µg g^−1^ of DOX of STR, respectively (Fig. 2). Therefore, these dosages were then used to treat *Brucella*-infected mice.

### Establishment of a chronic steady phase of infection

**Fig 3:**
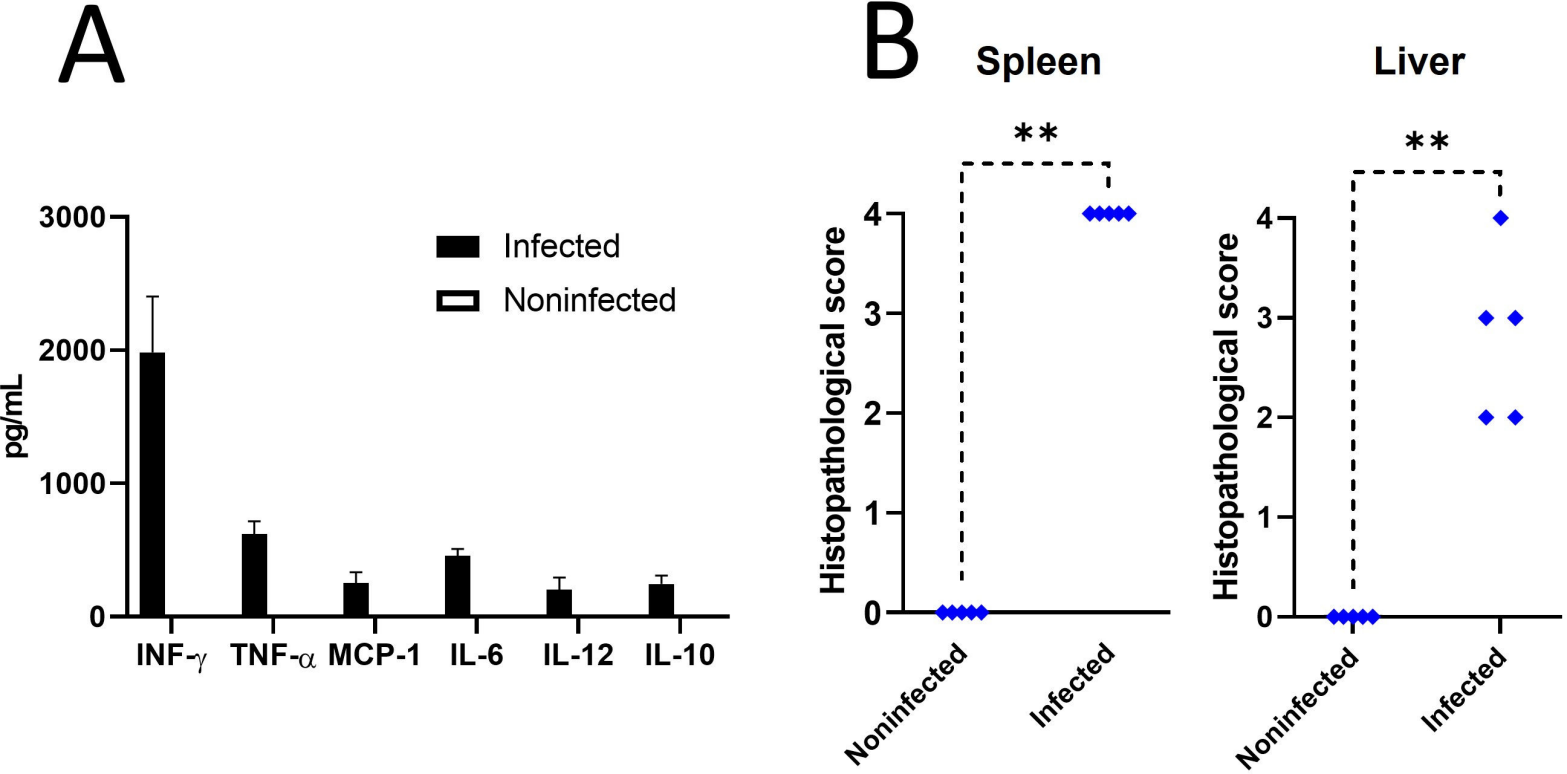
Cytokine quantification and histopathological score of *B. abortus* infected mice. Mice were infected with 10^4^ CFU of *B. abortus* 2308. After 30 days, (**A**) the levels of INF-γ, TNF-α, MCP-1, IL-6, IL-12, and IL-10 were quantified in plasma, and (**B**) the histopathological score was evaluated in hematoxylin eosin-stained spleen and liver sections. The *P*-value of <0.001 (**) is indicated.

As described, the chronic steady phase in the mouse model occurs from the third to the eighth to 11th week post-infection ()(47). Accordingly, we infected mice and waited until day 30 to reach this phase. Mice were evaluated at this time. As expected (Table 1), infected mice displayed significant changes in the hematological profile compared to noninfected mice. These changes included a decrease in leukocytes and lymphocytes and an increase in neutrophils and monocytes. In addition, infected mice showed an increased cytokine production (Fig. 3A) and a high histopathological score in the spleen and the liver, which are all expected at this chronic steady phase (Fig. 3B).

**TABLE 1:** Hematological values of noninfected and *B. abortus*-infected CD1 mice before antibiotic treatment at day 30 after infection^*a*^

Parameter	Unit	Noninfected		Infected	
		Mean	SD^*b*^	Mean	SD^*b*^
Leucocytes	10⁹/L	10.63	1.05	7.75 (*)	1.91
Lymphocytes	10⁹/L	8.26	1.42	2.90 (**)	1.43
Monocytes	10⁹/L	0.24	0.15	0.64 (**)	0.19
Neutrophils	10⁹/L	1.58	0.45	4.23 (**)	0.75
Lymphocytes	%	80.44	8.08	35.34 (**)	10.43
Monocytes	%	2.32	1.46	8.34 (**)	2.24
Neutrophils	%	15.06	4.18	56.34 (**)	8.93
Red blood cells	10^12^/L	11.22	0.52	11.49	1.04
Hemoglobin	g/dL	16.95	0.59	13.86 (**)	1.39
Hematocrit	%	64.03	11.37	51.40 (*)	5.39

^*a*^ *P* values <0.05 (*) and <0.01 (**) are indicated.

^*b*^ SD, standard deviation.

### Mice evaluation after antibiotic treatment

Hematological analysis performed after antibiotic treatment was completed in all experimental groups (Table 2). Significantly few changes were observed compared to the noninfected group. Both treated groups (infected and noninfected) showed an increase in lymphocytes. Meanwhile, the infected nontreated group showed a significant increase in neutrophils.

**TABLE 2:** Hematological values of mice subjected to different protocols at day 78 after *B. abortus* infection^*a*^

Parameter	Unit	Noninfected nontreated		Noninfected treated		Infected nontreated		Infected treated	
		Mean	SD^*b*^	Mean	SD^*b*^	Mean	SD^*b*^	Mean	SD^*b*^
Leucocytes	10⁹/L	6.23	1.83	7.89	2.06	10.67	4.57	9.79	4.77
Lymphocytes	10⁹/L	3.19	1.59	6.68	1.95	6.95	3.93	7.98 (*)	3.40
Monocytes	10⁹/L	0.50	0.61	0.22	0.18	0.57	0.34	0.21	0.19
Neutrophils	10⁹/L	0.94	0.40	0.96	0.19	3.10 (*)	1.89	1.60	1.33
Lymphocytes	%	82.08	0.84	84.16	3.95	64.20	20.36	84.20	6.31
Monocytes	%	2.00	1.64	5.52	4.71	14.44	18.93	2.00	1.36
Neutrophils	%	15.53	2.30	12.84	3.17	30.92	19.97	13.80	5.73
Red blood cells	10^12^/L	10.53	0.54	10.04	0.55	10.63	0.60	10.58	0.20
Hemoglobin	g/dL	15.93	0.73	15.34	0.67	14.66	1.62	16.16	0.48
Hematocrit	%	59.46	5.58	53.86	1.97	55.34	6.72	55.72	3.30

^*a*^ *P* values <0.05 (*) and <0.01 (**) are indicated.

^*b*^ SD, standard deviation.

**Fig 4:**
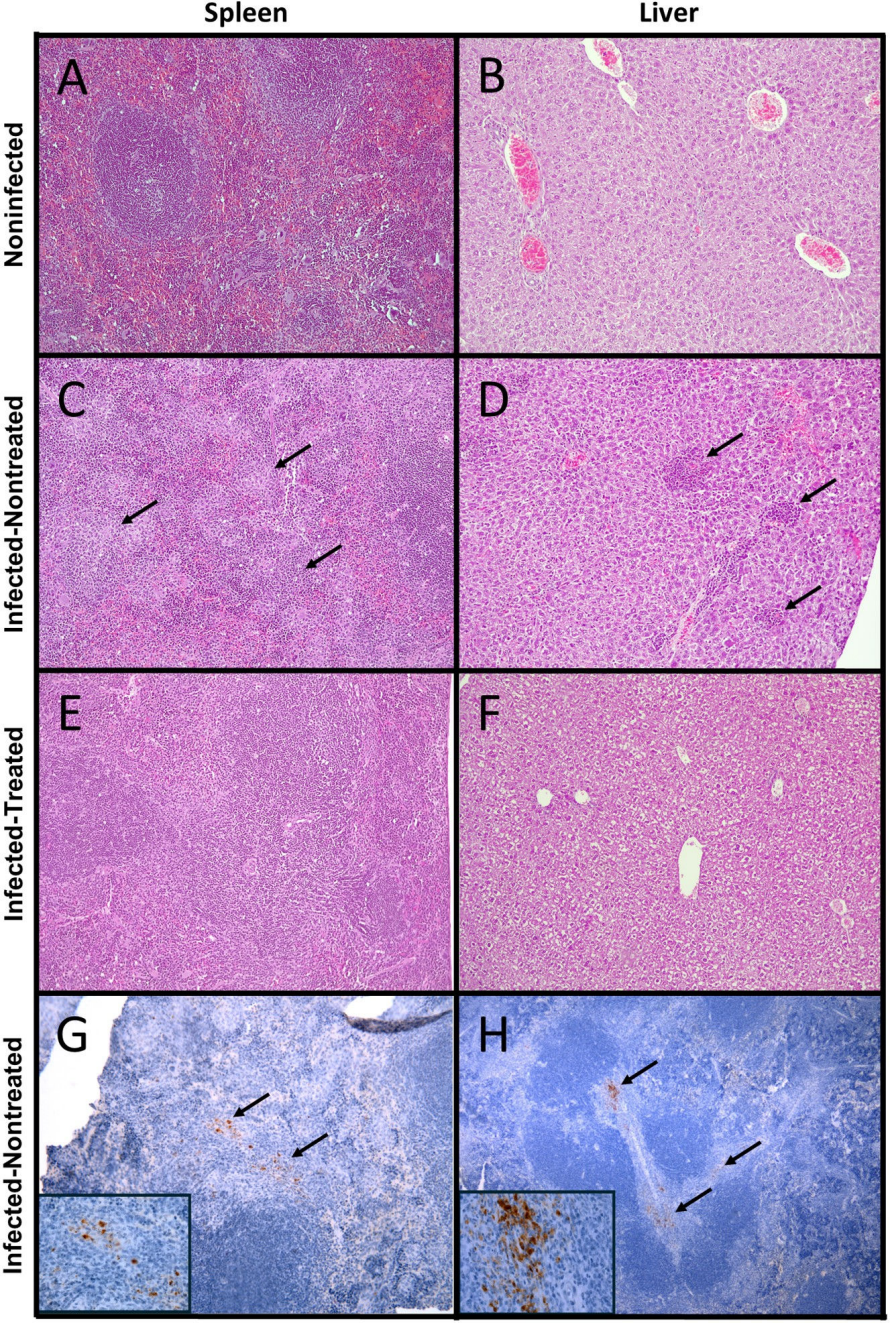
Histopathological analysis of *B. abortus* infected-treated mice. Mice were infected with 10^4^ CFU of *B. abortus* 2308. After 30 days post-infection, mice were either untreated with antibiotics (infected-nontreated), treated with STR (7.5 µg g^−1^) for 15 days and DOX (100 µg g^−1^) for 45 days (infected-treated). At 78 days post-infection, histopathological analysis was evaluated in hematoxylin eosin-stained spleen and liver sections (**A–F**). Arrows indicate the presence of granulomas. Immunoperoxidase detection of *Brucella* organisms (anti-lipopolysaccharide) in the red pulp (**G**) and white pulp around the central arteriole and marginal zone (**H**) of the spleen of mice at 30 days post-infection. Counterstaining was performed with Harris-hematoxylin. The arrows in G and H indicate the sites where the immunoperoxidase-staining bacteria are localized. The inserts correspond to the amplification of the immunostaining cells pointed by the upper arrow in the G and H panels.

**Fig 5:**
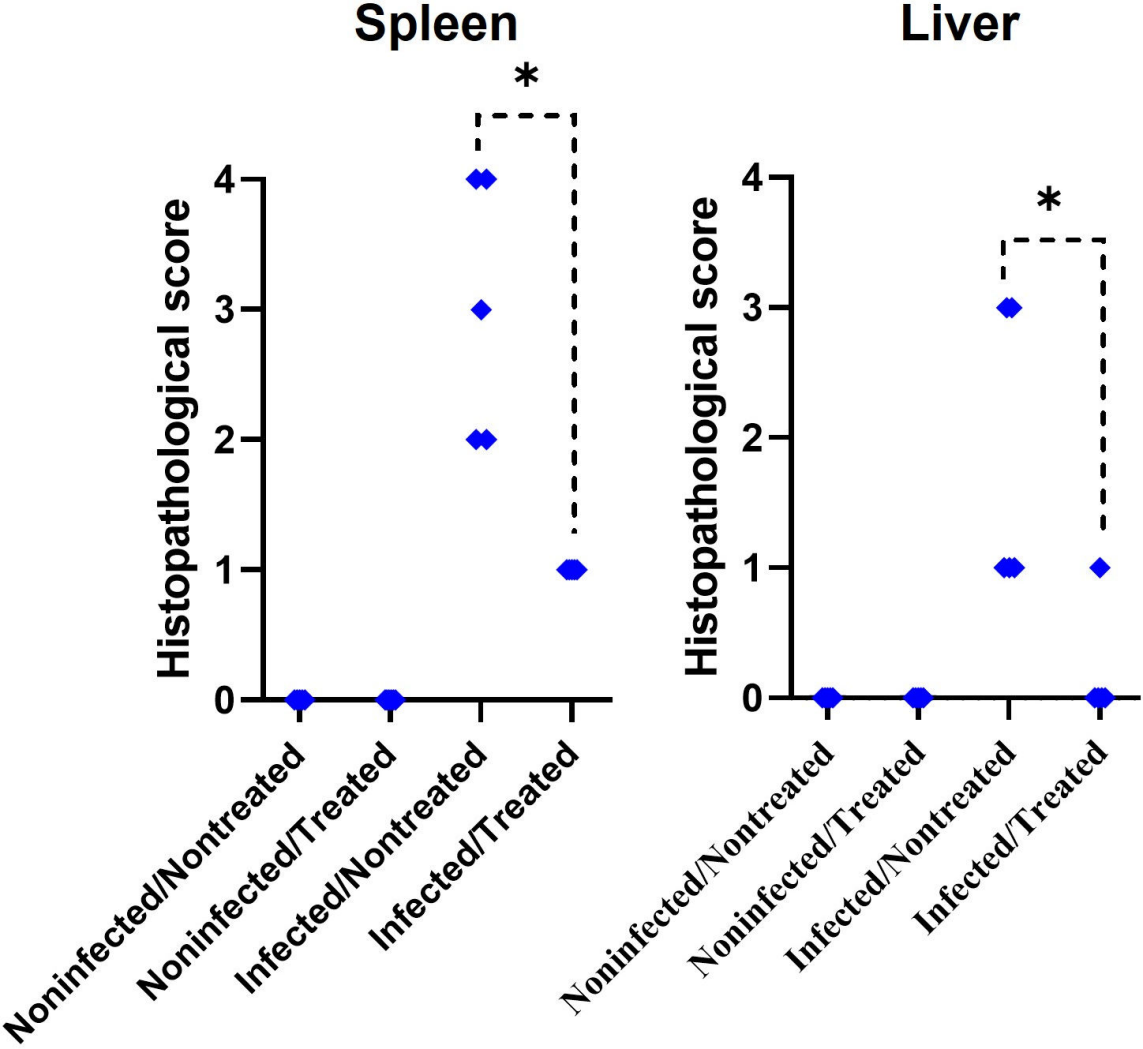
Histopathological score post-treatment. Mice were infected with 10^4^ CFU of *B. abortus* 2308. After 30 days post-infection, mice were treated with STR (7.5 µg g^−1^) for 15 days and DOX (100 µg g^−1^) for 45 days. At 78 days post-infection, the histopathological score was evaluated in eosin-stained spleen and liver sections. The *P*-value of <0.01 (*) is indicated.

**Fig 6:**
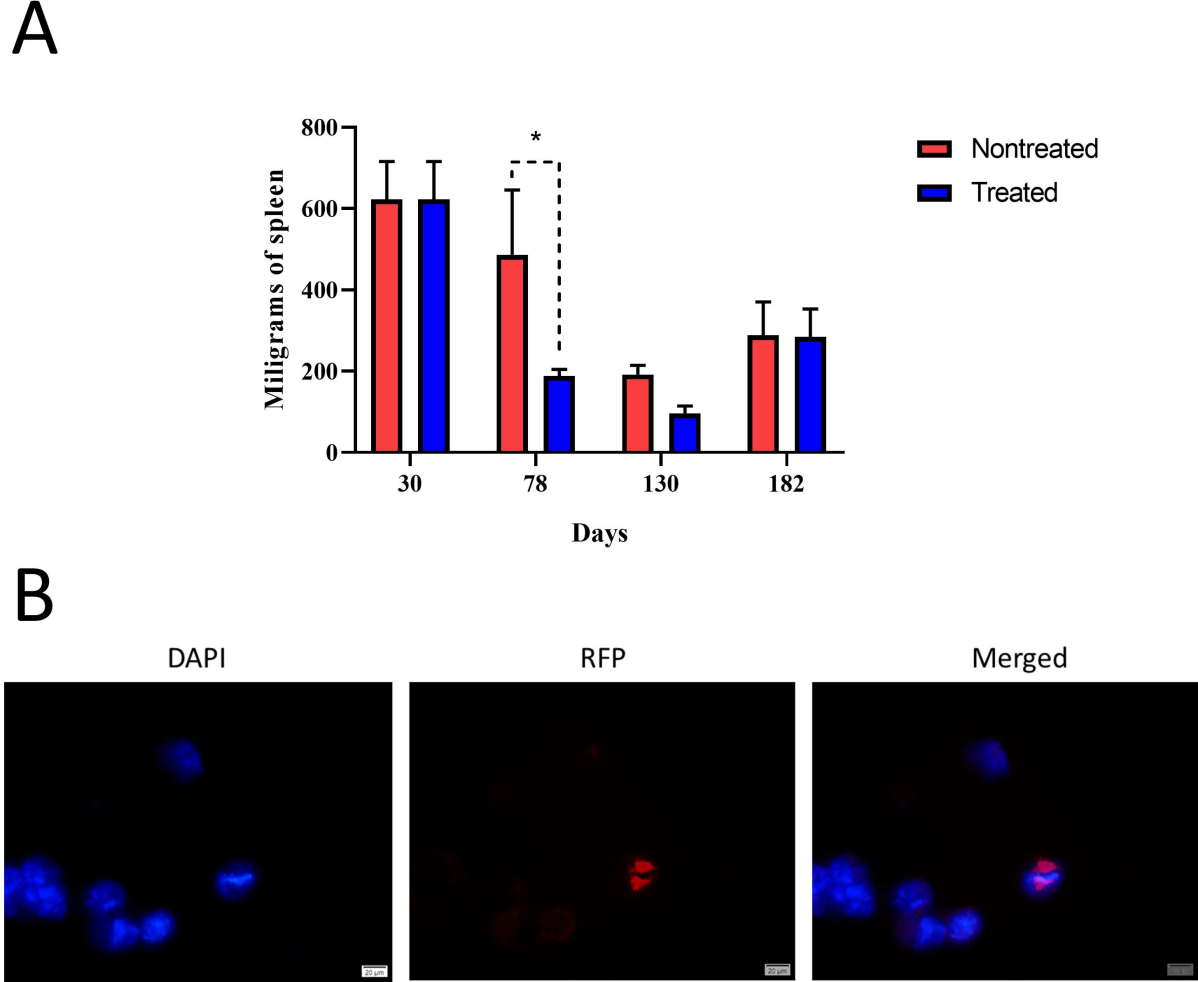
Spleen weights and fluorescent microscopy. Mice were infected with 10^4^ CFU of *B. abortus* 2308. After 30 days post-infection, mice were treated with STR (7.5 µg g^−1^) for 15 days and DOX (100 µg g^−1^) for 45 days. At 30, 78, 130, and 183 days post-infection, (**A**) spleen weight and (**B**) fluorescent microscopy in bone marrow cells were performed. Microscope images are at 400× magnification. Representative cells with DAPI-stained nuclei (blue) and intracellular fluorescent *B. abortus* RFP (red) were photographed under the microscope using the appropriate color filter channel. Images were cut from the microscope field, contrasted, saturated, and merged using Photopea Online Photo Editor. The *P*-value of <0.01 (*) is indicated.

In comparison to noninfected mice (Fig. 4A and B), histopathology images in infected-nontreated mice (Fig. 4C and D) showed typical brucellosis lesions characterized by splenomegaly, hepatomegaly, neutrophilic infiltrates, microgranulomas, and bacterial colonization, all consistent with a *Brucella* chronic infection. These characteristic lesions were significantly decreased in the infected/treated group in both tissues (Fig. 4E and F). Immunoperoxidase staining revealed *Brucella* organisms localized intracellularly in the spleen’s red and white pulp (Fig. 4G and H). These histopathological differences were quantified. Infected-treated mice also showed a significant decrease in the histopathological score compared to infected-nontreated mice (Fig. 5). Concomitantly, to the extent of infection, inflammation of the spleen (determined as spleen weight) was significantly higher in the infected-nontreated mice than in the antibiotic-treated infected group, except for the last point, at the time of relapses (Fig. 6A). Notably, noninfected-treated mice showed no pathological histopathological lesions, indicating that the treatment did not induce tissue changes potentially associated with prolonged antibiotic treatment.

Kidney and liver function were also evaluated in plasma (Table 3). Compared to noninfected mice, creatinine values increased in all groups, and ureic nitrogen increased in the infected/treated group, indicating a mild impairment in kidney function. ALT and AST liver enzyme values were raised in the infected-nontreated group. AST was also increased in the infected-treated group, suggesting some liver toxicity. Despite this, total protein production was not affected in any of the groups, as seen in the stability of total protein values and albumin.

**TABLE 3:** Evaluation of kidney and liver function of mice subjected to different protocols at day 78 after *B. abortus* infection^*a*^

Parameter	Unit	Noninfected		Noninfected treated		Infected nontreated		Infected treated	
		Mean	SD^*b*^	Mean	SD^*b*^	Mean	SD^*b*^	Mean	SD^*b*^
Ureic nitrogen	mg/dL	18.45	0.99	18.4	1.71	20.04	2.09	20.92 (*)	1.34
Creatinine	mg/dL	0.03	0.02	0.09 (*)	0.02	0.11 (**)	0.03	0.09 (**)	0.02
ALT^*c*^	U/L	49.25	4.44	48.00	2.53	112.8 (*)	74.80	67.40	18.77
AST^*d*^	U/L	135.25	3.11	141.00	18.54	200.8 (*)	67.27	192.2 (*)	51.28
Total protein	g/dL	6.15	0.25	5.76	0.14	6.44	0.43	6.22	0.33
Albumin	g/dL	3.55	0.11	3.40	0.06	3.30	0.21	3.56	0.14
Globulins	g/dL	2.60	0.21	2.38	0.07	3.14	0.62	2.66	0.24
Relation A/G		1.35	0.11	1.44	0.05	1.10	0.28	1.38	0.12

^*a*^ *P* values <0.05 (*) and <0.01 (**) are indicated.

^*b*^ SD, standard deviation.

^*c*^ ALT, alanine transaminase.

^*d*^ AST, aspartate aminotransferase.

### The course of bacterial loads, antibody titer, and cytokine quantification

Infected mice were treated with STR and DOX from day 31 to day 75 (Fig. 1). On days 78 and 137 post-infection, CFU counts in the spleen, liver, and bone marrow were undetectable in the treated group (Table 4). Likewise, qPCR and immunofluorescence analysis of the organs were also negative in all organs at this lapse. However, on day 182 post-infection, *Brucella* was detected in different organs of the antibiotic-treated group, indicating relapses (Table 4). We used bacteriological culture and qPCR from the culture-enriched bacterial liquid media to maximize Brucella detection. In cases where cultures or qPCR were negative, we used immunofluorescence in bone marrow (Table 5). Detection of bacteria by immunofluorescence after the relapse was not straightforward and required a significant scanning of the microscopic fields to detect just a few positive-infected cells (Fig. 6B). While in some cases, all organs were positive, in others, only one or two were positive, indicating the relevance of sampling different tissues.

**TABLE 4:** *B. abortus* detection in mice before and after DOX and STR treatment

Day	DOX + STR treatment	*B. abortus* isolation						Rate of infection
		Spleen		Liver		Bone marrow		
		Ratio	CFU/g^*a*^	Ratio	CFU/g^*a*^	Ratio	CFU/g^*a*^	
30	Pre-T^*b*^	5/5	6.7 × 10^6^ ± 3.7 × 10^6^	5/5	3.6 × 10^6^ ± 3.1 × 10^6^	5/5	7.9 × 10^5^ ± 3.9 × 10^5^	100%
78	Post-T^*c*^	0/5	n.d.^*d*^	0/5	n.d.^*d*^	0/5	n.d.^*d*^	0%
130	Post-T^*b*^	0/7	n.d.^*d*^	0/7	n.d.^*d*^	0/7	n.d.^*d*^	0%
182	Post-T^*c*^	3/7	5.7 × 10^4^ ± 2.8 × 10^4^	6/7	6.3 × 10^3^ ± 1.9 × 10^3^	2/7	1.2 × 10^4^ ± 1.0 × 10^4^	100%^*e*^

^*a*^ Mean values CFU/gram of tissue ± Error Standard.

^*b*^ Pre-T, pre-treatment.

^*c*^ Post-T, post-treatment.

^*d*^ n.d., non-detected.

^*e*^ At least one organ of the seven mice was infected with *B. abortus*.

**TABLE 5:** Detection of *B. abortus* in different tissues according to the used technique after relapses at day 182 of infection^*a*^

Treatment	Mice number	Tissue					
		Spleen		Liver		Bone marrow^*b*^	
		CFU/g^*c*^	qPCR^*d*^	CFU/g^*c*^	qPCR^*d*^	CFU/g^*c*^	qPCR/IF
PBS (45 days)	1	1.0 × 10^3^	qPCR	5.9 × 10^2^	n.d.	n.d.	n.d.
	2	n.d.	n.d.	n.d.	n.d.	n.d.	n.d.
	3	2.9 × 10^5^	n.d.	4.8 × 10^4^	n.d.	n.d.	qPCR
	4	4.1 × 10^4^	n.d.	7.7 × 10^4^	n.d.	1.4 × 10^5^	n.d.
	5	7.0 × 10^5^	qPCR	1.8 × 10^3^	qPCR	n.d.	n.d.
	6	1.9 × 10^5^	n.d.	4.7 × 10^3^	n.d.	6.6 × 10^4^	qPCR+IF
STR 7.5 µg/g (15 days) and DOX 100 µg/g (45 days)	1	6.7 × 10^4^	n.d.	7.8 × 10^3^	n.d.	1.3 × 10^4^	IF^*e*^
	2	3.8 × 10^3^	n.d.	5.9 × 10^2^	n.d.	n.d.	n.d.
	3	n.d.	n.d.	2.8 × 10^3^	n.d.	n.d.	n.d.
	4	n.d.	n.d.	6.5 × 10^3^	n.d.	n.d.	n.d.
	5	1.5 × 10^5^	n.d.	1.4 × 10^4^	n.d.	1.1 × 10^4^	IF^*e*^
	6	n.d.	n.d.	n.d.	qPCR	n.d.	n.d.
	7	n.d.	n.d.	2.8 × 10^3^	n.d.	n.d.	qPCR

^*a*^ n.d., non-detected.

^*b*^ IF was only performed in bone marrow.

^*c*^ CFU/gram of tissue.

^*d*^ qPCR, positive quantitative polymerase chain reaction.

^*e*^ IF, positive immunofluorescence.

**Fig 7:**
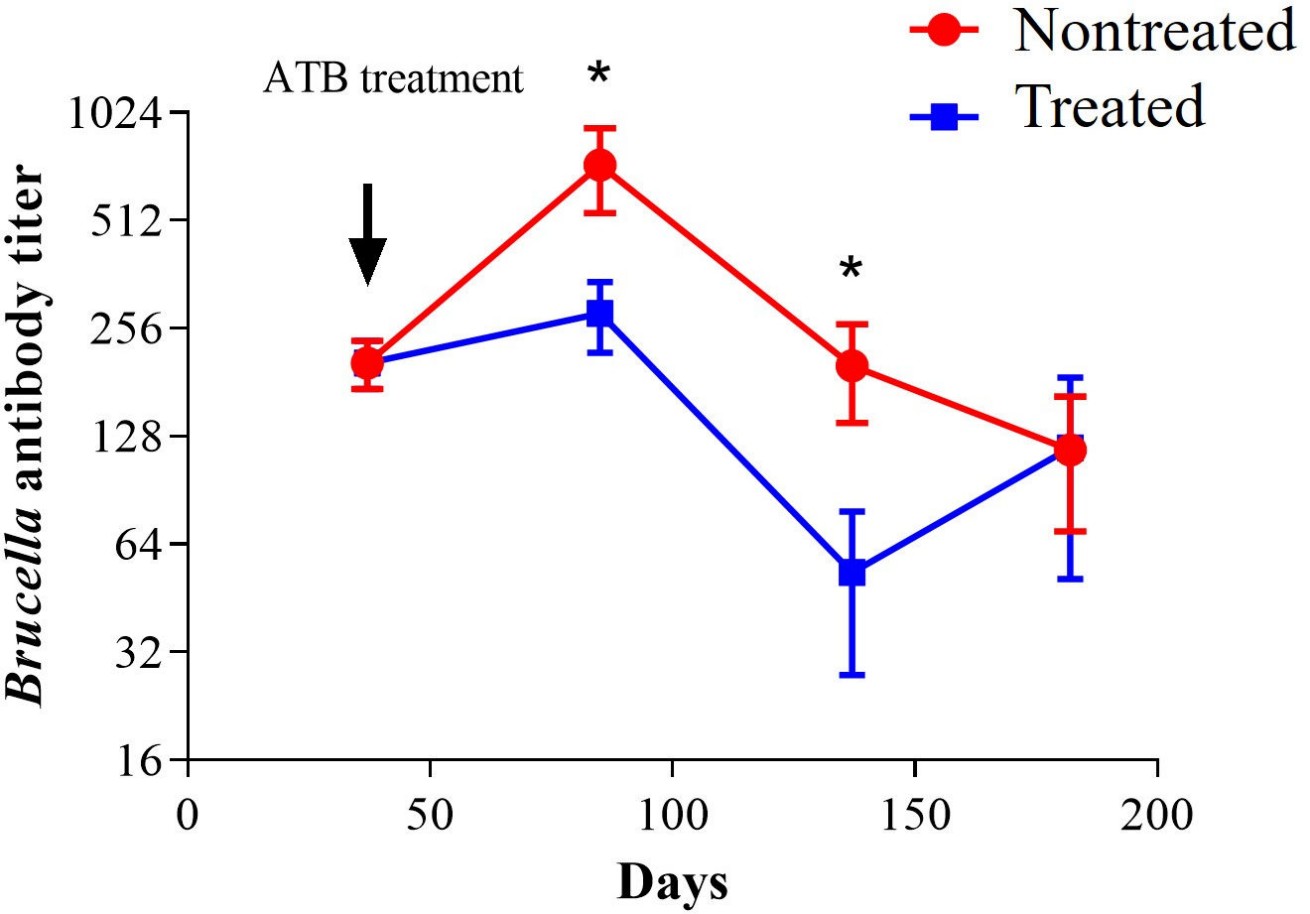
Anti-*Brucella* antibody titers. Mice were infected with 10^4^ CFU of *B. abortus* 2308. After 30 days post-infection, mice were treated with STR (7.5 µg g^−1^) for 15 days and DOX (100 µg g^−1^) for 45 days. At 30, 78, 130, and 183 days post-infection, the *Brucella* antibody titers were quantified by microagglutination in a 96-well plate. The *P*-value of <0.01 (*) is indicated.

**Fig 8:**
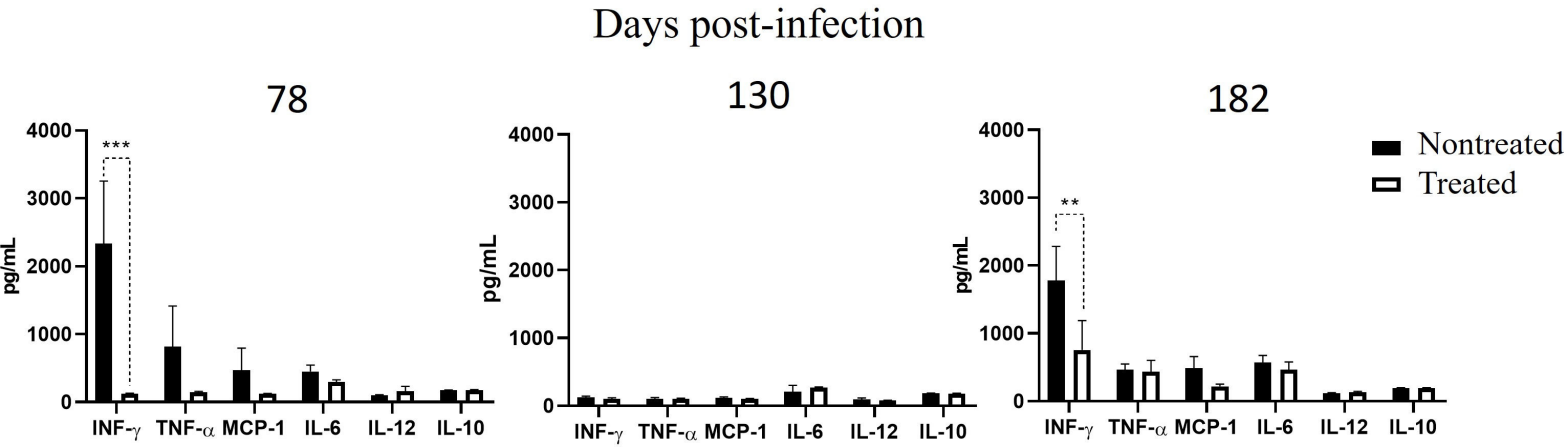
Cytokine quantification in infected-treated mice. Mice were infected with 10^4^ CFU of *B. abortus* 2308. After 30 days post-infection, mice were treated with STR (7.5 µg g^−1^) for 15 days and DOX (100 µg g^−1^) for 45 days. At 78, 130, and 183 days post-infection, the levels of INF-γ, TNF-α, MCP-1, IL-6, IL-12, and IL-10 were quantified in serum. The *P*-values of <0.001 (**) and <0.0001 (***) are indicated.

*Brucella* detection in the infected-treated group was associated with increased spleen weight (Fig. 6A). Likewise, *Brucella* antibody titers in the antibiotic-treated group significantly decreased until day 137. However, after this period, relapses appeared, and antibodies increased, reaching the same level depicted by the untreated group (Fig. 7A). At day 78 post-infection (3 days after antibiotic treatment finished), a significant decrease in INF-γ was observed in the antibiotic-treated group (Fig. 8). Unexpectedly, at day 130 post-infection, the low level of cytokines in the nontreated group paralleled that of the antibiotic-treated group. When infection reappeared on day 182, cytokines increased again in the antibiotic-treated group; however, INF-γ (the insignia cytokine in brucellosis) levels were significantly lower compared to the untreated group (Fig. 8).

All bacteria recovered in the relapses displayed the same MIC sensitivity to DOX and STR as the parental strain used for infecting mice. Moreover, short-read WGS analysis was performed to compare the reference strain used for infection with one isolate from the untreated group and one isolate from the treated group. An SNP (G/A) in position 2,324,388 of the alignment; 200,140 of the chromid (smaller chromosome) was found in one isolate from the infected/treated group. It corresponds to a synonymous substitution in a gene coding for a glutathione-regulated potassium-efflux system protein (locus tag BRUAB_RS11375).

## DISCUSSION

The most efficient method for treating human brucellosis with short to medium-term evolution is a combination of DOX and STR. In complications such as endocarditis, spondylitis, meningoencephalomyelitis, abscesses, and other focal forms, generally, a more severe antibiotic regime with three antibiotics for a longer time is required (1, 8, 12, 48). Due to the *Brucella* organisms’ low or nil antibiotic resistance appearance (14), R/TF can be treated again following the same antibiotic regimes with higher chances of success (1, 9). The causes of brucellosis recurrence in antibiotic-treated patients remain elusive. Some risk factors associated with R/TF are the premature suspension of the antibiotics, less-effective antibiotic therapy, positive blood cultures in the initial stages of the disease, 10 days or more disease duration before treatment, male sex, low platelet counts (8) (8, 49) the more virulent *B. melitensis* causes more R/TF than *B. abortus* or other *Brucella* strains (50). Of note is that the clinical features presented by those individuals who relapse are commonly less severe compared to the brucellosis symptoms displayed before antibiotic treatment (1, 8, 51). This condition may be due to the acquired immunity after the initial infection.

Our findings parallel the antibiotic treatment of human brucellosis. For instance, like in humans, it was observed in mice that the number of *Brucella*-positive cultures rapidly declined after the first weeks of treatment, with the accompanying decrease of the critical cytokine levels and resolution of organ focal infections (48, 52, 53). Likewise, mice’s hematological values and kidney and liver functions are compatible with those reported in humans after antibiotic therapy (54). Likewise, the level of anti-*Brucella* antibodies in antibiotic-treated infected mice dropped (48, 55). However, it is also true that some individuals remain with high antibody titers for a protracted period after treatment (48, 56, 57). Anamnestic responses due to renewed bacterial contacts cannot be ruled out because some individuals may come from brucellosis-endemic areas. As in mice, the human R/TF (48, 58) was characterized by positive bacterial cultures, increasing cytokine levels, and augmented anti-*Brucella* antibodies. The rise of cytokine levels observed in both treated and untreated mice 182 days post-infection may indicate a cyclical or wave-like pattern of cytokine production during chronic infection. While not directly assessed in this study, it is plausible that these fluctuations could result from the persistent nature of the infection.

Measuring antibody levels to determine the course of antibiotic treatment in brucellosis may have some value, mainly when sera has been obtained during the disease and the treatment regime. However, in other cases, it is unpractical since it is difficult to establish a reliable threshold that can differentiate between active infection, remission, or R/TF. In contrast, measuring the level of cytokines, in particular, INF-γ, the key cytokine in brucellosis, seems to be a more reliable parameter to follow the success of antibiotic therapy (52) and to detect R/TF as a sign of bacterial proliferation (59, 60).

One apparent difference between the experimental murine model and natural human brucellosis is that the number of relapses was 100% for the former. The R/TF after DOX + STR treatment seldom exceeds 5% in humans (7, 8, 61, 62). However, in contrast to the diagnoses carried out in humans, the sensitivity of the murine model increased the odds of identifying “relapses” through exhaustively exploring three target organs and using three different methods for bacterial detection. It has been proposed that in human brucellosis, the chances of bacterial isolation increase by up to 50% if, in addition to blood, the bone marrow is included in *Brucella* organisms’ detection (63, 64). Nevertheless, this is not straightforward, and others have not confirmed this claim (65–67).

We do not know which precise day bacteria initiated replication during relapses in the 52–130 and 182-day window. In clinical trials conducted during and immediately after treatment, *Brucella* has been isolated in some asymptomatic patients. These asymptomatic “bacteriological relapses” have been observed during the first few weeks of antibiotic treatment (68), a phenomenon known since the 1950s (3, 69). It would be necessary to narrow the bacterial isolation within the 52-day window and determine if a second round of antibiotics (DOX + STR) or an extension of the treatment with lower amounts of antibiotics (e.g., monotherapy with DOX) would be adequate to prevent R/TF.

The average number of *Brucella* in the blood during the bacteremia peak in humans has been estimated at less than 85 CFU/mL (70), which, compared to the number of cells in the blood, is minuscule. Consequently, the DNA host contamination from the biological samples ranges billions of times more than the *Brucella* DNA extracted by regular procedures. Moreover, *Brucella* organisms are difficult to disrupt, and stringent methods using substances that interfere with the qPCR assay are required (71). Therefore, qPCR and immunofluorescence remain as presumptive second-line complementary assays for the presence of *Brucella* organisms, which always require confirmation by direct culture methods (18, 72).

We ignore if the presence of *Brucella* after antibiotic treatment is as ubiquitous in humans as in mice. Based on the persistence of DNA detection after successful treatment, some authors speculate that *Brucella* organisms remain hidden in the body for long periods after treatment without clinical symptoms and that R/TF may appear several months or even years after treatment (73). It may be that the bacterium endures hidden within cells without obvious clinical symptoms and, for a protracted period, not detected by culturing methods. Like in mice, in long-lasting human forms of the disease, *Brucella* organisms have been detected in bone marrow cells (74, 75). Although the bone marrow pathology in most patients ameliorates or disappears after antibiotic treatment (76), bacteria may remain hidden in cells without symptoms, as revealed by the transmission of brucellosis after bone marrow transplants from an asymptomatic donor (77).

It has been shown that although antibiotic therapy cleared >99.5% of the *Salmonella*, the treatment was inefficient against a *Salmonella* subset residing in the white pulp of the spleen, where the density of neutrophils and monocytes required for bacterial clearance is lower than in other spleen compartments (78). Previously, we have shown that *Brucella* is resistant to the killing action of neutrophils, hampering their function (6), and that, as shown here, *Brucella* also resides in the white pulp in the spleen. Whether this is relevant for *Brucella* latent infections remains to be proved.

It has been extensively documented that *Brucella* infects a significant number of professional and non-professional phagocytes (5, 79) and that its particular location within cisterns of the endoplasmic reticulum protects the bacterium from antibiotics (5, 80, 81). Likewise, it has been determined that the *Brucella* cell envelope, particularly the lipopolysaccharide molecule, confers protection against bactericidal substances, including some antibiotics (24). Other proposed mechanisms comprise efflux pumps (82), type II Toxin-Antitoxin systems (83), and the formation of a subpopulation of bacteria that transiently displays an antibiotic-tolerance phenotype without the need for genetic modification (62). A combination of all these proposed mechanisms may likely contribute to the persistence of the bacterium in tissues despite prolonged antibiotic treatments.

In this study, *Brucella* organisms recovered during relapses showed the same MIC previously determined for DOX and STR before infection. Additionally, the WGS analysis did not reveal changes that could explain the treatment failure, reinforcing the notion that the emergence of antibiotic resistance in *Brucella* organisms is rare, as previously noted (15, 84). Therefore, the emergence of *Brucella* antibiotic resistance field strains, mainly rifampicin (85, 86) and other antibiotics (15, 84), must be taken cautiously. Indeed, the MICs for *Brucella* organisms have not been strictly determined, and a distinction between the strains’ *in vitro and in vivo* antibiotic susceptibility must be established. Lower MICs may be helpful in long-lasting brucellosis if administered for sufficient time. Accurately determining the MIC for organisms such as *Brucella* is challenging due to their intracellular habitat. Similarly, controls such as the rifampicin-resistant strain, *B. abortus* RB51, with a defined mutation encoded in the rpoB region, are generally omitted in the concomitant MIC assays. Accordingly, the emergence of chromosomal-coded resistant strains should be confirmed by genetic characterization and not just by MIC assays.

Critical questions for resolving R/TF in brucellosis lie in finding the most suitable antibiotics, doses, and administration regimes that achieve bactericidal concentrations in the target organs where the *Brucella* reside and in cases where brucellosis exhibits clinical complications. Previously, we developed a suitable method for detecting the DOX and STR concentrations in the target organs (28). Following this, we have determined the optimal bactericidal concentration levels in the organs of mice, which are at least four times the *in vitro* MIC for DOX and STR. Consequently, the proportional doses used in mice and humans differ. Indeed, the corresponding daily doses of 200 and 1,000 mg of DOX and STR, respectively, recommended for humans (average weight of 70 kg), which are equivalent to ~3 and ~14 µg g^−1^ of DOX and STR, respectively (1), depart from the daily optimal doses for mice (100 and 7.5 µg g^−1^ for DOX and STR, respectively). However, it is unlikely that the proportion of relapses observed in mice and humans was due to these differences since the doses used in mice of DOX and STR were 30 times higher and half lower, respectively.

Moreover, the critical concentrations of these antibiotics in the organs for treating human brucellosis are unknown. Following 200 mg of DOX orally and 500 mg of STR intravenously in adults, the averaged peak serum levels at 24 h of DOX (~1.5 µg g^−1^) (87) and of STR (~2.8 µg g^−1^) (88), respectively. Although they are in a similar range, the antibiotic values achieved in humans are lower than in the murine model (~7.5 and ~4 µg g^−1^ of DOX and STR, respectively, after 12 h of administration).

Achieving therapeutic concentrations was insufficient to eliminate *Brucella* organisms from the mouse body. However, increasing antibiotic dosage poses challenges due to the risk of kidney and liver toxicity, particularly with STR. We observed a mild decrease in kidney function and detectable liver damage in treated animals. It has been suggested that a monotherapy regimen of DOX lasting 45 days can produce good results, avoiding parenteral administration of STR, which entails higher cost, toxicity, and difficulty of application in rural areas where brucellosis occurs more frequently; though, R/TF are more likely to occur (89, 90). Exploring this alternative in our experimental model would be worthy of establishing suitable monotherapy conditions.

We ignore whether the observations regarding the presence of bacteria in the organs of mice after antibiotic treatment are idiosyncratic of our model or correspond to a more general phenomenon also happening in humans. Despite its limitations, when appropriately established, the murine model alerts us of the possible outcomes and helps to bring general conclusions to understand human brucellosis.
